# Marizomib Promotes Senescence or Long-Term Apoptosis in Melanoma Cancer Cells

**DOI:** 10.3390/molecules29235652

**Published:** 2024-11-29

**Authors:** Wiktoria Monika Piskorz, Rafał Krętowski, Marzanna Cechowska-Pasko

**Affiliations:** Department of Pharmaceutical Biochemistry, Medical University of Bialystok, Mickiewicza 2A, 15-222 Białystok, Poland; wiktoria.piskorz@sd.umb.edu.pl (W.M.P.); rafal.kretowski@umb.edu.pl (R.K.)

**Keywords:** proteasome inhibition, senescence, apoptosis, cancer treatment, salinosporamide A, NPI-0052

## Abstract

Cellular senescence is cell cycle arrest and the inhibition of cell proliferation. New anticancer approaches include the elimination of cancer cells through the induction of senescence followed by senolysis. New prosenescence compounds are still being searched for. Little is known about the ability of proteasome inhibitors to induce senescence in tumor cells, especially in malignant melanoma. The aim of our study was to verify the activity of a natural proteasome inhibitor—marizomib (MZB)—directly after incubation and after its removal to assess its potential to induce senescence or long-term apoptosis in malignant melanoma cell lines (A375 and G361). After 48 h of incubation with MZB, we observed an increased number of SA-β-galactosidase-positive cells, upregulated expression of P21 and P-P53 proteins and an increased number of cells at the subG1 phase (line G361) or at both the subG1 and G2/M phases (line A375). After 96 h from inhibitor removal, the G361 line presented signs of senescence (increased level of SA-β-galactosidase, IL-8, P-P53, G2/M and S phases of cell cycle, decreased lamin B1 and cleaved lamin B1), while the A375 line demonstrated more signs of apoptosis (increased subG1 phase, P-P53, cleaved lamin B1). The gathered findings suggest that MZB resulted in the induction of cellular senescence (line G361) or enhanced apoptosis (line A375) in the melanoma cell lines tested here and could be a promising therapeutic factor in malignant melanoma treatment.

## 1. Introduction

Malignant melanoma is a skin cancer that originates from melanocytes. It is characterized by extremely dynamic growth and the highest mutation frequency of all types of cancers [1,2]. The basic treatment of melanoma includes surgical excision, which is sufficient in the case of skin melanomas in the radial growth phase [3]. However, a serious problem occurs in the case of metastases, which are often difficult to detect and develop extremely dynamically [4]. In such cases, chemotherapy, radiotherapy, immunotherapy or targeted therapy are used. Chemotherapy involves the use of drugs that inhibit proliferation or lead to the death of cancer cells. In the past, chemotherapeutic agents such as alkylating agents (i.e., temozolomide), taxanes (like paclitaxel or docetaxel) or platinum agents, including cisplatin and carboplatin, were used in the treatment of melanoma [5]. With the development of anticancer strategies, targeted therapies using BRAF and MEK inhibitors as well as immunotherapies with the use of immune check point inhibitors, like cytotoxic T lymphocyte-associated antigens 4 (CTLA-4), programmed cell death 1 (PD-1), PD ligand 1 (PDL-1) and lymphocyte-activation gene 3 (LAG3), have been included in the fight against melanoma [6,7]. However, all these strategies share the same problems: resistance to treatment and serious side effects [8,9]. For this reason, it is important to search for new solutions that are both effective and safe. The reports on the supportive effects of vitamin D supplementation in melanoma therapy are noteworthy [7,10]. There are also promising reports on the use of oncolytic viruses [11,12] or mRNA vaccines against melanoma [13,14]. The fight against malignant melanoma requires new, effective and safe strategies. One of them may be using a natural proteasome inhibitor—marizomib (MZB)—to induce cellular senescence or apoptosis in malignant melanoma cells.

Cellular senescence is a process that inhibits cell division by arresting the cell cycle [15]. Maintenance of this state depends on cyclin-dependent kinases (CDKs) inhibitors—P21WAF1/CIP1 (CDKN1A) and P16INK4A (CDKN2A)—regulated by the retinoblastoma protein (RB) and P53 protein.

There are three phases of senescence: primary senescence, developing senescence and late senescence [16]. The P53 protein may be responsible for the induction of apoptosis, inhibition of the cell cycle or initiation of senescence. In primary senescence, as a result of DNA damage response (DDR), P53 activates the cyclin-dependent kinase inhibitor P21. The effect of both proteins is the inhibition of the cell cycle [17]. In the developing senescence state, P21 and P53 are still active, and the P16 pathway may also be activated. Inhibition of CDKs by P16 and P21 prevents RB hyperphosphorylation. This allows RB to form a complex with E2F and inhibit the cell cycle. Additionally, morphological changes in the cells and senescence-associated secretory phenotype (SASP) release occur. In the late senescence state, the P21 pathway is decreased, and the cell cycle arrest is maintained by the P16 pathway and by the overproduction of SASP. This state is also indicated by the presence of SA-beta galactosidase [16]. It has been proven that the induction of senescence in cancer cells is possible. Several methods to induce this process have been reported, including telomerase inhibition, topoisomerase inhibition, epigenetic modulation, cell cycle modulation (CDK inhibitors), P53 targeting and, potentially, proteasome inhibition [18,19]. However, using senescence in anticancer therapy may have negative consequences. In the long term, senescent cells may create a tumor-promoting microenvironment [20]. It has been reported that senescence-associated secretory phenotype (SASP) can promote epithelial–mesenchymal transition (EMT) [21,22,23], immunosuppression [22,24], chemoresistance [25,26,27], acceleration of cancer progression [28,29] and invasiveness [30,31]. In order to mitigate these deleterious effects, senolytics—drugs eliminating senescent cells—are used. The combination of senescence promotors and senotherapeutics appears to be a promising strategy to fight cancer [32,33,34,35,36]. Although the efficiency of this method depends on many factors, the selectivity and effectiveness of presented trials demonstrate the importance of these studies for future cancer therapy. Further research on currently known senolytics and prosenescent drugs, and the search for new ones, is essential.

One strategy to promote cancer cell senescence could potentially be proteasome inhibition. The proteasome is an enzyme complex involved in the regulated degradation of proteins. It is composed of 28 subunits arranged in four rings: two outer ones, made of α subunits—responsible for proteolytic selectivity—and two internal ones, made of β subunits, which are proteolytically active [37]. It is involved in maintaining cellular homeostasis, controlling signal transduction and maintaining the cell cycle, cell division and stress response [38]. Proteasome activity is also favorable in cancer progression. Accelerated tumor growth—associated with aberrant RNA splicing, genetic mutations and enhanced protein synthesis—contributes to the production of misfolded proteins. In order to maintain cancer cell viability, overproduced and damaged proteins must be eliminated by proteasome [39]. It has been proven that proteasome inhibitors block the β subunits of the 20s proteasome, effectively inactivating survival pathways and increasing endoplasmic reticulum (ER) stress [40]. Under physiological conditions, protein kinase RNA-like endoplasmic reticulum kinase (PERK) is inactivated, because it is associated with the immunoglobulin heavy chain-binding protein (BiP), also known as 78 kDa glucose-regulated protein (GRP78). During ER stress, BiP/GRP78 proteins dissociate from PERK. PERK autophosphorylation occurs, which leads to the phosphorylation of eukaryotic initiation factor 2 alpha (EIF2α). EIF2α phosphorylation can lead to different outcomes. The first one is an activation of activating transcription factor 4 (ATF4) and subsequently C/EBP homologous protein (CHOP), leading cells to the apoptosis pathway [41]. The second one is the induction of GADD45α, which may cause cell cycle arrest in the G2/M phase, suggesting ongoing senescence [42]. Moreover, research indicates that some proteasome inhibitors have the ability to induce premature aging of human skin fibroblasts [43,44,45,46] and cervical cancer cells [46].

In our previous studies on marizomib, we observed an increase in the level of phosphorylated EIF2α, BIP and CHOP proteins in malignant melanoma cells treated with the inhibitor. This confirmed the thesis that apoptotic changes that occurred in treated cells were related to ER stress [47]. In the current research, we wanted to check whether proteasome inhibition-mediated senescence occurs in malignant melanoma cell lines A375 and G361 after the addition of marizomib (MZB) (Salinosporamide A; NPI-0052), a natural proteasome inhibitor derived from marine bacteria [48]. Marizomib is considered to have a broad spectrum of activity due to its potent and sustained inhibition of all three proteolytic activities of the proteasome [49,50] and appears to be well tolerated by the organism [51]. Marizomib has been reported to be effective as an anticancer compound in the treatment of several types of cancer [49,50]. However, its ability to induce senescence in cancer cells, particularly melanoma cells, has not yet been determined. In our research, we aimed to investigate the potential of marizomib to induce senescence in the A375 and G361 melanoma cell lines.

The aim of our study was to check whether the activity of marizomib against malignant melanoma cells is also associated with the induction of cellular senescence. We believe that the fight against cancer should be approached in a multi-pronged manner. Previous studies have shown us that marizomib has the ability to induce apoptosis in malignant melanoma cells, but a certain part of the population does not undergo these changes. However, those cells might develop a senescence phenotype and may be targeted by senolytic drugs. We believe that studying cellular senescence, especially in combination with senolysis, may influence the development of new anticancer strategies.

## 2. Results

### 2.1. The Effect of Marizomib on SA-β-Galactosidase Expression

**Figure 1:**
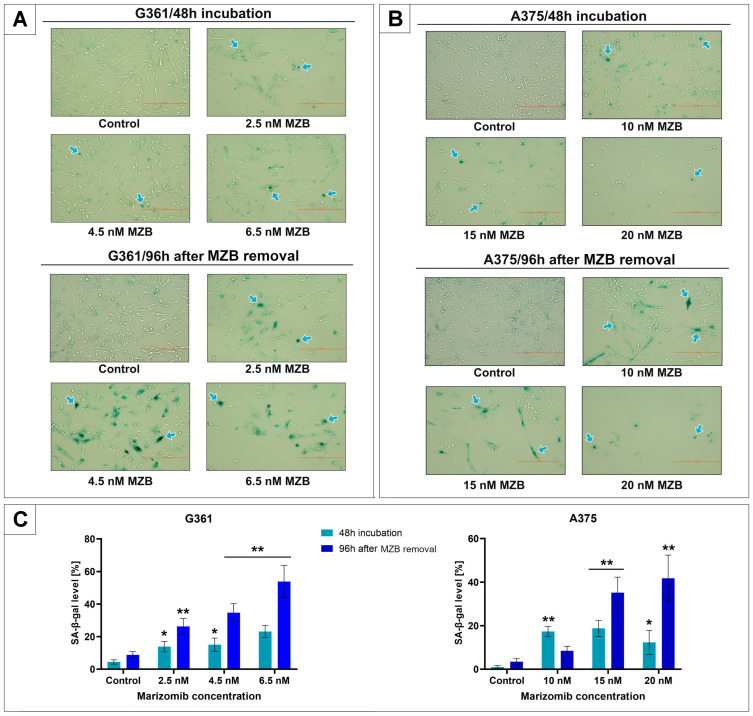
The effect of marizomib (MZB) on SA-β-galactosidase expression in A375 and G361 melanoma cell lines. The G361 line was incubated with MZB concentrations ranging from 2.5 to 6.5 nM and the A375 line was incubated with MZB concentrations ranging from 10 to 20 nM for 48 h. Some of the cells were stained with x-gal substrate directly after the initial 48 h incubation. In the remaining samples, the medium was replaced with fresh one, without MZB, and the cells were left for 96 h at 37 °C and 5% CO_2_. After this time, staining was performed. The SA-β-galactosidase-positive cells were stained blue. Examples of positive cells are indicated by arrows (panel **A**,**B**). Subsequently, cells were counted, and the percentage of positive cells (panel **C**) is presented in the bar graphs. Mean values from three independent experiments ± SD are shown. Significant alterations are expressed relative to each control and marked with asterisks. Statistical significance was considered if * *p* < 0.05; ** *p* < 0.001.

The senescence-promoting activity of marizomib (MZB) was evaluated by SA-β-galactosidase staining with x-gal substrate. Cells were exposed to increasing concentrations of marizomib (line G361: 2.5–6.5 nM; line A375: 10–20 nM) for 48 h. Some of the samples were stained immediately after 48 h of incubation with MZB. In the rest, the MZB medium was replaced with fresh medium, without the addition of inhibitor, and the cells were incubated for another 96 h at 37 °C and 5% CO_2_. SA-β-galactosidase-positive cells were stained blue (Figure 1A,B). The percentage of positive cells at each concentration is presented in bar graphs (Figure 1C). MZB increased SA-β-galactosidase expression in a concentration-dependent manner. The most pronounced rise in the G361 line was visible at a concentration of 6.5 nM MZB, where the percentage of positive cells reached 23.16 ± 3.71%. After 96 h following the 48 h incubation with MZB, this value increased further to 53.84 ± 9.18%. In the A375 line, the highest increase in SA-β-galactosidase expression after 48 h was observed at 15 nM MZB and amounted to 18.1 ± 3.69% of positive cells. A total of 96 h after the 48 h incubation with MZB, this percentage rose to 35.24 ± 7.08%. Moreover, the percentage of positive cells treated with 20 nM MZB also increased significantly over time and reached 41.8 ± 10.66%.

### 2.2. The Effect of Marizomib on Lamin B1 and Interleukin 8

**Figure 2:**
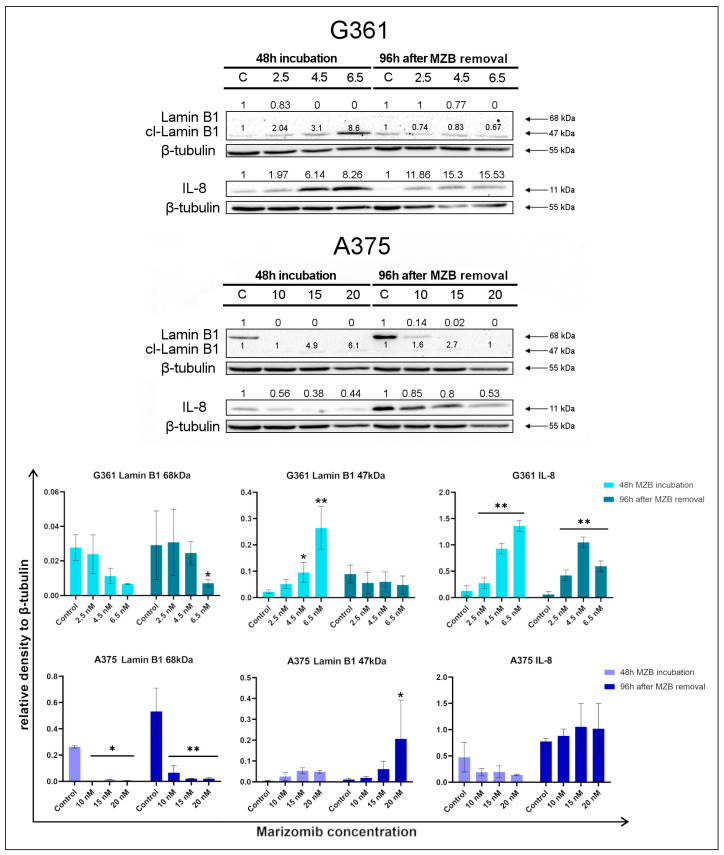
Western blot analysis of lamin B1 and interleukin 8 (IL-8) expression in marizomib-treated G361 and A375 melanoma cell lines. Cells were incubated with different MZB concentrations for 48 h. Some of the cells were examined immediately after incubation. In the rest, the medium was changed to fresh, without inhibitor. Cells were left for 96 h at 37 °C and 5% CO_2_ and then examined. The representative bands of the lamin B1, IL-8 and β-tubulin are illustrated. Time of incubation and concentrations used (in nanomoles) are presented below each cell line name. Samples containing 15 μg of protein were submitted for electrophoresis and immunoblotting. Densitometric analysis is presented above the bands as relative fold change in comparison to untreated controls (C). The expression of each untreated control was set as 1. β-tubulin expression (55 kDa) was used as a loading control. Bar graphs present densitometric analysis as relative to β-tubulin density. Significant changes are expressed relative to each control and are marked with asterisks. Mean values from three independent experiments ± SD are presented. Statistical significance was considered if * *p* < 0.05; ** *p* < 0.001.

Lamin B1 is a nuclear intermediate filament protein involved in maintaining cell proliferation [52]. As a result of the activation of the P53 pathway, lamin B1 is lost. Reducing the amount of this protein (68 kDa) is one of the markers of cellular senescence [53]. Lamin B1 may also indicate the process of apoptosis, during which it is cleaved by caspases. Cleaved lamin B1 is visible on Western blot analysis at the level of 40–50 kDa [54]. In order to check the expression of lamin B1 in the MZB-treated melanoma cells, Western blot analysis was performed (Figure 2). In the G361 line, lamin B1 with a mass of 68 kDa was slightly visible in the control samples and decreased with increasing marizomib concentration, which indicates the ongoing senescence process. The increase in the signal of cleaved lamin B1 (47 kDa) was much more pronounced after 48 h incubation with MZB, suggesting the ongoing process of apoptosis. A total of 96 h after MZB removal, the level of cleaved lamin B1 decreased slightly compared to the control sample. In the A375 line, the 68 kDa lamin B1 signal was more prominent than in the G361 line, and its level decreased with increasing marizomib concentration. The signal from cleaved lamin B1 (47 kDa) was weaker, but the expression of this protein increased both after 48 h MZB incubation and after 96 h following MZB removal.

Interleukin 8 (IL-8) overexpression occurs in late-senescent cells. Western blot analysis of IL-8 expression (Figure 2) revealed that after 48 h incubation with MZB, the level of this protein in the G361 line increased, while in the A375 line, IL-8 expression did not change significantly. A similar situation occurred after 96 h following MZB removal.

### 2.3. The Effect of Marizomib on Cell Cycle

**Figure 3:**
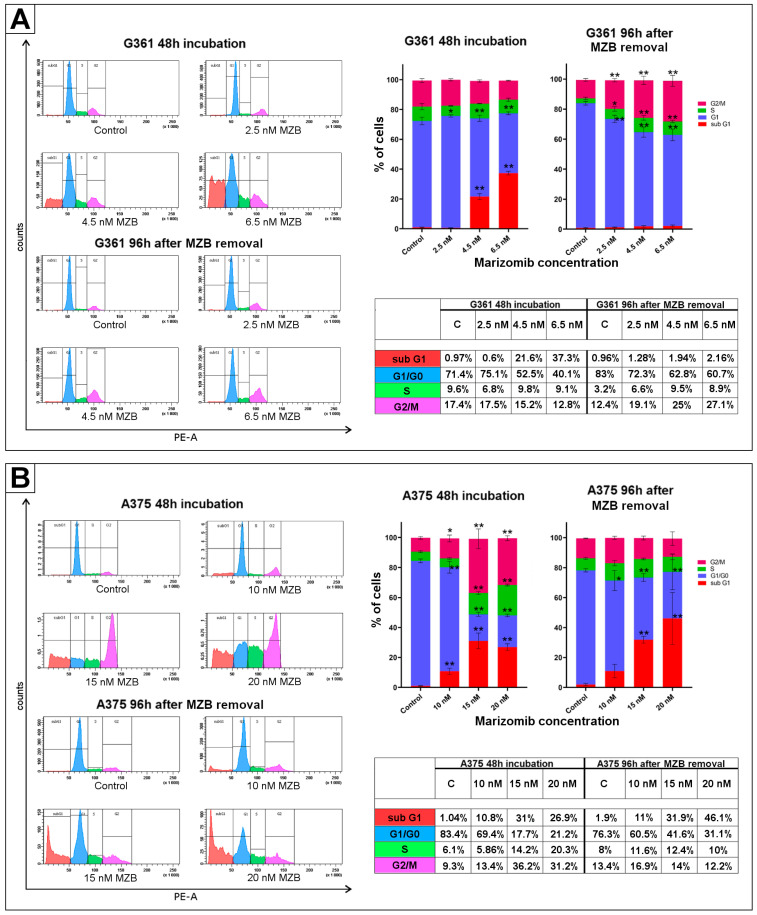
The effect of marizomib (MZB) on melanoma cell cycle distribution. Cell cycle was evaluated by propidium iodide staining followed by flow cytometry analysis. The G361 melanoma cells were treated with MZB concentrations from 2.5 to 6.5 nM, while the A375 cells were treated with 10–20 nM for 48 h. After this time, some of the cells were submitted for analysis. In the rest, the culture media was replaced with fresh media without MZB, and cells were incubated for 96 h at 37 °C, 5% CO_2_. Subsequently, the analysis was performed. Histograms (panels **A**,**B** on the **left**) present cell cycle profiles obtained with flow cytometry, and bar graphs (panels **A**,**B** on the **right**) show the cell cycle distribution in percents. Tables (panels **A**,**B** on the **right**) present the average percentage of cells of each phase, depending on the time of incubation and the concentration of MZB. Significant changes are expressed relative to each control and are marked with asterisks. Mean values from three independent experiments ± SD are presented. Statistical significance was considered if * *p* < 0.05; ** *p* < 0.001.

In order to analyze the effect of MZB on the cell cycle of the examined melanoma cells, a flow cytometry assay was performed. The concentrations of MZB used in the experiment ranged from 2.5 to 6.5 nM in the G361 line and from 10 to 20 nM in the A375 line. Cells were incubated with the inhibitor for 48 h. Some of the cells were submitted to analysis immediately after incubation. In the rest, the culture media were replaced with fresh media, without MZB, and cells were further incubated for 96 h at 37 °C, 5% CO_2_. After this time, analysis was performed. The collected results confirmed the effect of the inhibitor on the melanoma cell cycle (Figure 3); however, it appears to affect cells differently depending on the cell line. In the G361 line, after 48 h of incubation with the inhibitor, an increase in the apoptotic subG1 phase was observed, which reached an average of 37.3% at a concentration of 6.5 nM MZB. At the same time, the G1/G0 phase decreased and reached the lowest average value of 40.1% in the 6.5 nM MZB. Cell distribution was noticeably different after 96 h following the 48 h of incubation with MZB. After the 96 h period, researched cells exhibited signs of cell cycle arrest in the S and G2/M phases. At a concentration of 6.5 nM, an increase in S-phase cells to an average of 8.9% and an increase in G2/M phase to an average of 27.1% were observed. Furthermore, the G1/G0 phase decreased to an average of 60.7%, which is higher than after 48 h of incubation. Additionally, in contrast to the cells examined immediately after 48 h of incubation, no significant changes were observed in the subG1 phase. All the percentages are presented in the following tables (Figure 3A,B). In the A375 line, after 48 h incubation with MZB, a significant increase in the G2/M, subG1 and S phases was observed. The highest average percentage of cells at the subG1 (31%) and G2/M (36.2%) phases was recorded at a concentration of 15 nM MZB, while the highest number of cells at the S phase (20.3%) was observed at 20 nM MZB. Furthermore, the percentage of cells at the G1/G0 phase declined significantly in the presence of the inhibitor and reached an average of 17.7% at 15 nM MZB. The distribution of cell cycle phases changed after 96 h from the withdrawal of MZB. There were no more signs of S and G2/M phase inhibition. However, a decrease in the G1/G0 phase was visible, which reached an average of 31.1% at 20 nM. At the same time, an increase in the subG1 phase also intensified and reached an average of 46.1%. All percentages are presented in the tables (Figure 3A,B).

**Figure 4:**
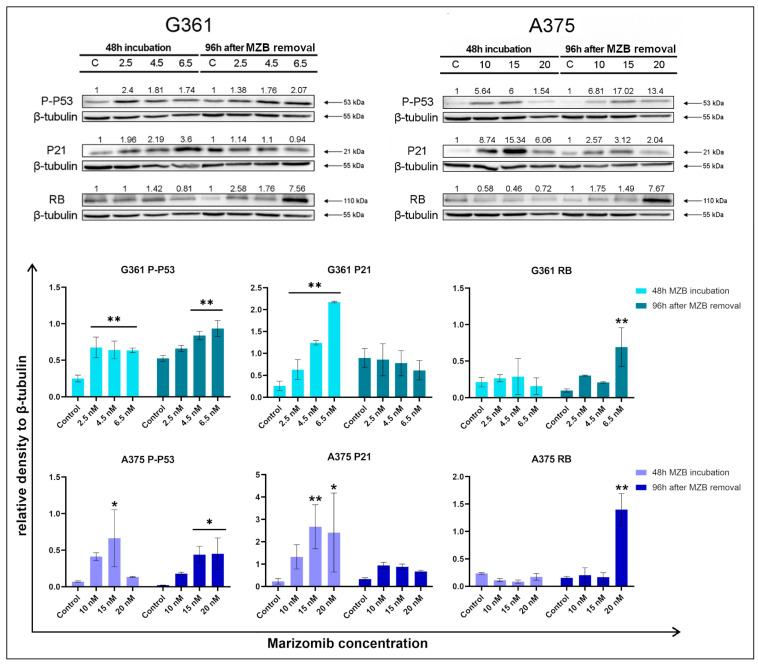
Western blot analysis showed the expression of P-P53, P21 and RB proteins. The representative bands of P-P53, P21, RB and β-tubulin are illustrated. Time of incubation and concentrations used (in nanomoles) are presented below each cell line name. Densitometric analysis is presented above the bands as a relative fold change compared to each untreated control (C). The control expression level was set as 1. β-tubulin (55 kDa) expression was used as a loading control. Samples containing 15 µg of protein were submitted to electrophoresis and immunoblotting. Bar graphs present densitometric analysis as relative to β-tubulin density. Significant changes are expressed relative to each control and are marked with asterisks. Mean values from three independent experiments ± SD are presented. Statistical significance was considered if * *p* < 0.05; ** *p* < 0.001.

Western blot analysis (Figure 4) of P21 and P-P53—proteins associated with the cell cycle—showed an increase in the expression of these proteins in marizomib-treated cells. In the G361 line, the signal form P-P53 was enhanced both directly after 48 h incubation and 96 h after MZB removal. In the A375 line, this tendency was similar. In both cell lines, after 48 h of incubation, the level of P21 protein increased strongly. After 96 h following MZB removal, P21 expression weakened. In both cell lines, the RB protein after 48 h of incubation remained at a similar level as in the control samples. However, after 96 h after the inhibitor’s removal, in both cell lines, the RB protein level clearly increased, reaching a maximum at the highest tested concentrations.

## 3. Discussion

Malignant melanoma is regarded as the most dangerous skin cancer. Due to the rapid development and frequency of metastases, as well as the lack of response to treatment, this cancer still remains a therapeutic challenge [1,4]. New methods of treatment are constantly being sought. One of these new methods is the induction of cellular senescence and subsequently the senolysis of cancer cells. Both senescence inducers and senolytics are widely investigated in various types of cancer. Attempts are still being made to discover or develop new compounds causing these processes. Currently known methods of senescence induction include topoisomerase inhibition, telomerase inhibition, epigenetic regulation and regulation of the cell cycle and P53 protein [18,19]. Proteasome inhibitors also seem to be a promising group, although their ability to induce senescence is poorly investigated, especially in cancer cells [43,44,45,46].

The aim of our study was to verify the ability of marizomib—a natural inhibitor that inhibits all three proteasome activities (caspase-like (C-L, β1), trypsin-like (T-L, β2), and chymotrypsin-like (CT-L, β5))—to induce cellular senescence in two malignant melanoma lines: A375 and G361. After performing a test for the expression of SA-β-galactosidase—one of the best-known markers of cellular aging—an increase in the number of SA-β-gal-positive cells was observed in samples treated with the inhibitor. This effect intensified over time even after MZB removal, which indicates a state of late senescence [16]. It is worth noting that 96 h after the 48 h incubation, in the G361 line, the level of SA-β-gal reached higher mean values than in the A375 line, even despite the lower concentration of MZB. Increased expression of SA-β-galactosidase due to the action of proteasome inhibitors was also observed by Chondrogianni et al. [43], Torres et al. [44] and Ukekawa et al. [45] when conducting research on fibroblasts. Furthermore, En et al. [46] showed an increase in SA-β-galactosidase due to the action of the proteasome inhibitor MG132 in HeLa cells.

In senescent cells, the expression of lamin B1 with a mass of 68 kDa decreases [53], which can be observed in both researched cell lines. Interestingly, in the G361 line, an increase in cleaved lamin B1 with a mass of 47 kDa was clearly visible only after 48 h of incubation, suggesting an ongoing apoptosis process. After 96 h following the removal of the inhibitor, the level of cleaved lamin B1 remained similar to the control, which may indicate weakening of the apoptosis process in favor of cellular senescence. In contrast to the G361 line, in the A375 line, the cleaved 47 kDa lamin B1 was slightly visible; however, its level increased relative to the control, especially 96 h after the removal of the inhibitor. The results in lamin B1 already indicate differences in the response to marizomib between cell lines. These differences become even more apparent when analyzing the cell cycle.

The cell cycle analysis we performed does not distinguish between living, capable of dividing cells and G1-arrested senescent cells, as both groups appear in the G1/G0 phase. However, this test provides a proper illustration of other changes occurring in treated cells. After 48 h of incubation with MZB, cells of both cell lines showed signs of apoptosis [47]. This is also confirmed by the increase in the subG1 phase we observed. However, 96 h after the withdrawal of the inhibitor, the subG1 phase in the G361 line remained close to the control level. In return, the number of G2/M and S phase cells increased, and the number of G1/G0 phase cells was higher than immediately after 48 h of incubation. Cell cycle analysis of the G361 line suggests that these cells underwent apoptosis (increase in subG1 phase) during 48 h of incubation with MZB. After the removal of the inhibitor, apoptosis decreased and the cells entered a state of cell cycle arrest in S, G2/M and probably G1/G0 phases. Considering the strong increase in P21 and P-P53 proteins after 48 h of incubation, there is much evidence that P21-related senescence occurs here. This is also confirmed by the increasing level of IL-8—one of the SASP factors.

According to research by Shtutman et al., a high level of P21 results in cell cycle arrest in the G1, S and G2/M phases and increased levels of SA-β-gal, which coincides with our results [55]. In P21-induced senescence, activation of the P21 protein is transient and decreases after stabilization of senescence. P16 is then responsible for maintaining senescence. Considering that the P16 protein is absent from the G361 and A375 cells, it is likely that, in this case, increased levels of P53 protein are responsible for maintaining senescence [16]. With insufficient levels of P53 protein, senescent cells may re-enter the cell cycle [56], while an excess of P53 directs cells towards apoptosis [57].

This mechanism may also explain the changes occurring in A375 cells. Here, similarly to the G361 line, after 48 h of incubation, apoptosis occurs [47], as indicated by an increase in the subG1 phase. The increase in the subG1 phase in the A375 line after 48 h of incubation with marizomib was also observed by Millward et al. [58]. The percentage of subG1 phase recorded by the researchers was about 40% in 10 nM MZB and 75% in 50 nM MZB, which is higher than in our studies, but we have also observed an increase in the S and G2/M phases. Moreover, 96 h after MZB withdrawal, the levels of the S and G2/M phases decreased to the control level, but the subG1 phase increased significantly. These changes suggest that, in the case of this cell line, MZB induces cellular stress and cell cycle inhibition associated with ER stress and activation of the P53/P21 pathway and subsequently directs cells towards apoptosis. The lack of significant changes in the level of IL-8 also contributes to this conclusion. Research indicates that significant amounts of P53 are required for apoptosis to occur, with relatively low levels of P21. Senescent cells are unable to accumulate enough P53, which is one of the reasons why they do not undergo apoptosis [59]. The A375 cells demonstrated a greater increase in P-P53 protein compared to the control than the G361 cells, which may be the reason for the observed late apoptosis. This phenomenon might partially explain the differences between cell lines in response to marizomib.

Chondrogianni et al. [60], in studies on fibroblasts, proved that the fate of fibroblasts treated with epoxomicin (a strong, selective and irreversible inhibitor of chymotrypsin-like activity) is dependent on the functioning of the P21/P53 and P16/RB pathways. According to the research of Chondrogianni et al. [60], partial inhibition of the proteasome due to the activity of the P21/P53 pathway and the lack of signal from the P16/RB pathway leads to G2/M cell cycle arrest and then to cell death, which is consistent with the results of our studies in the A375 line. However, the G361 line presented a different outcome. It is likely that, despite the activity of the P21/P53 pathway and P16 inactivation, the final response is also determined by the level of P53 protein.

**Figure 5:**
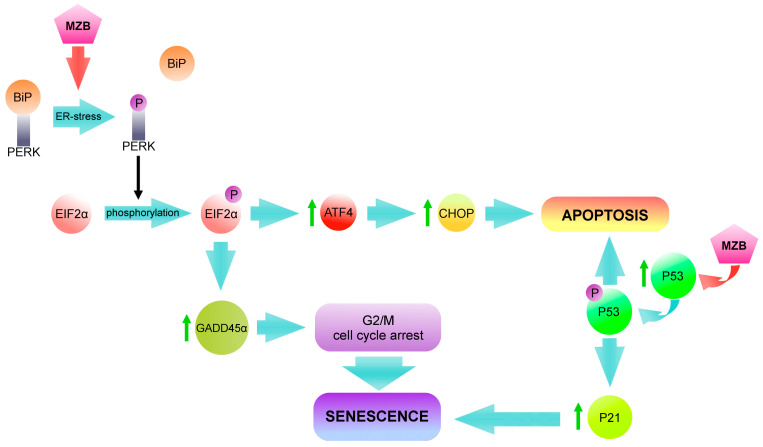
Under physiological conditions, protein kinase RNA-like endoplasmic reticulum kinase (PERK) is associated with immunoglobulin heavy chain-binding protein (BiP). ER stress activates BiP dissociation and PERK autophosphorylation, which leads to the phosphorylation of eukaryotic initiation factor 2 alpha (EIF2α). P-EIF2α can activate two pathways leading to different outcomes. The first upregulates the activating transcription factor 4 (ATF4) and, subsequently, the C/EBP homologous protein (CHOP), which directs cells to an apoptosis pathway. The second is the upregulation of GADD45α, which results in G2/M cell cycle arrest, suggesting ongoing senescence. Furthermore, phosphorylated P53 increases the expression of P21 protein, which prevents retinoblastoma phosphorylation and induces the senescence state. The fate of the cell depends to a large extent on the amount of P53 protein. An increased P53 level is needed to maintain senescence. The P16/RB pathway is also responsible for maintaining this state. Further significant overexpression of P53 may direct cells to the path of apoptosis. (Red arrows indicate the site of action of marizomib. Blue arrows represent the course of the reaction. Green arrows indicate an increase in protein levels.)

To summarize, our results indicate that marizomib induces ER stress in both malignant melanoma cell lines [47]. As a result, PERK and subsequently EIF2α undergo phosphorylation, and P-EIF2α can activate two pathways (Figure 5). The first one leads cells to apoptosis through the activation of ATF4 and CHOP [61]. The second one induces G2/M cell cycle arrest through GADD45α [42,62]. The effects of this pathway are visible immediately after 48 h incubation with MZB. On the other side, the P53/P21 pathway is activated, which initiates the senescence state. The results of this path become visible after time. After 96 h following the removal of the inhibitor, apoptosis deepens in the A375 line. This is indicated by the increase in the subG1 phase and cleaved lamin B1 level and the similar level of IL-8. Some cells undergo senescence (increased SA-β-gal and RB, decreased lamin B1), but they are in the minority compared to apoptotic cells. Cells of the G361 line, on the other hand, after 96 h following the withdrawal of MZB, demonstrate more signs of senescence. The level of SA-β-gal, RB and IL-8 increases, while lamin B1 decreases. The number of G2/M and S phase cells also increases. The observed difference in the response of A375 and G361 cells may be due to the level of P-P53 protein, which is elevated enough in the G361 cells to maintain senescence but not high enough to induce apoptosis. In contrast, the expression of P-P53 protein in the A375 cells may be sufficient to initiate the apoptosis process.

We believe that the fight against cancer should be approached in a multi-pronged manner. Our previous studies have shown that marizomib has the ability to induce apoptosis in malignant melanoma cells, but a certain part of the population does not undergo these changes. Current research suggests that, after some time from removing MZB, the apoptosis process may deepen, or cells may enter a senescent state. It should be borne in mind that senescent cells and their secretory phenotype may be unfavorable in cancer therapy. In such a case, senolytic therapy should be considered. We believe that combining marizomib with senolytic therapy may give interesting results in the fight against malignant melanoma.

### Study Limitations

Our studies were conducted on two malignant melanoma cell lines: A375 and G361. We are aware of how diverse cancers originating from the same tissues can be. Unfortunately, based on our studies, we are unable to determine how marizomib will affect other melanoma cell lines. Moreover, in the in vitro model, the cells are exposed to the inhibitor for a fixed period of time, and the metabolism of other cells and tissues does not affect them. Therefore, at this stage, we are unable to determine what the activity of marizomib will be in the human body.

## 4. Materials and Methods

### 4.1. Reagents

Dulbecco’s modified Eagle’s medium (DMEM), containing glucose (4.5 mg/mL) with GlutaMaxTM, fetal bovine serum Gold (FBS Gold), trypsin-EDTA, phosphate-buffer saline (PBS), penicillin and streptomycin were provided by Gibco (San Diego, CA, USA). McCoy’s 5A Medium (ATCC 30-2007) was obtained from American Type Culture Collection (ATCC). BCA Protein Assay Kit and PierceTM ECL Western blot Substrate were provided by Thermo Scientific (Rockford, IL, USA). RIPA buffer and Protease/Phosphatase Inhibitor Cocktail were obtained from Cell Signalling Technology (Boston, MA, USA). Immuno-Blot PVDF Membranes for Protein Blotting were provided by Bio-Rad (Hercules, CA, USA). Antibodies provided by Cell Signalling Technology (Boston, MA, USA) include P21 Waf1/Cip1 (12D1) rabbit mAb, Phospho-P53 (Ser15) rabbit antibody, lamin B1 (D9V6H) rabbit mAb and β-Tubulin rabbit antibody. Antibodies provided by ABclonal (Woburn, MA, USA) include RB (A17005) rabbit pAb and IL8 (A2541) rabbit pAb. Marizomib was a product of MedChemExpress (New York, NY, USA). Senescence Detection Kit (ab65351) was provided by Abcam (Cambridge, UK). Ethanol was a product of POCH (Gliwice, Poland).

### 4.2. Cell Cultures and Exposure to Marizomib

Malignant melanoma cell lines: G361 (ATCC CRL-1424; mutant genes: BRAF, CDKN2A, STK11) and A375 (ATCC CRL-1619; mutant genes: BRAF, CDKN2A) were obtained from American Type Culture Collection (ATCC). A375 cells were cultured in DMEM, while G361 cells were cultured in McCoy’s 5A Medium, both supplemented with 10% heat-inactivated fetal bovine serum Gold (FBS Gold), penicillin (100 U/mL) and streptomycin (100 µg/mL) in accordance with the manufacturer’s recommendations. Cells were cultured in an incubator Galaxy S+ (RS Biotech, Irvine, UK) at 37 °C in an atmosphere enriched with 5% CO_2_. Confluent cells were rinsed with DPBS, detached with 0.05% trypsin and 0.02% EDTA then counted in a Scepter Cell Counter (Millipore, MA, USA) and seeded in plates for 24 h. Subsequently, the media was replaced with fresh media containing different concentrations of marizomib (2.5; 4.5; 6.5 nM in the G361 line and 10; 15; 20 nM in the A375 line). Cells were incubated for 48 h at 37 °C in an atmosphere enriched with 5% CO_2_. After this time, some of the cells were subjected to measurements. For the rest, the medium with the inhibitor was replaced with fresh medium without the inhibitor. Cells were then incubated for 96 h at 37 °C, with 5% CO_2_ and then subjected to measurements.

### 4.3. SA-β-Galactosidase Staining

The A375 and G361 cells were seeded in 6-well plates (2.5 × 10^5^ cells per well) in 2 mL of medium and left for 24 h at 37 °C with 5% CO_2_. Cells were then treated with different concentrations of marizomib (the A375 line: 10 nM; 15 nM; 20 nM; and the G361 line: 2.5 nM; 4.5 nM; 6.5 nM) for 48 h. Some of the cells were analyzed immediately after incubation with MZB, while in the rest, MZB was removed and cells were left for 96 h at 37 °C, 5% CO_2_. Subsequently, cells were washed with 2 mL of PBS (pH 7.4). SA-β-galactosidase-positive cells were detected using a Senescence Detection Kit (Abcam). Cells were fixed with 1 mL of Fixative Solution for 10 min and then washed twice with 2 mL of PBS. The Staining Solution Mix was prepared (940 µL of Staining Solution, 10 µL of Staining Supplement and 50 µL of 5-bromo-4-chloro-3-indolyl-β-d-galactopyranoside (x-gal) at 20 mg/mL concentration on each well) and added at pH 6.0. Appropriate pH ensures that non-senescent cells remain unaffected. The cells were incubated overnight at 37 °C without CO_2_, then washed with PBS, photographed and counted.

### 4.4. Cell Cycle Assay

Melanoma cells were seeded in a 6-well plate (2.5 × 10^5^ cells per well) and subsequently exposed to different concentrations of marizomib (the A375 line: 10–20 nM, the G361 line: 2.5–6.5 nM) for 48 h. Some of the cells were submitted to analysis immediately after MZB treatment. In the rest, the MZB medium was replaced with fresh medium without the inhibitor, and cells were left for 96 h at 37 °C and 5% CO_2_. After incubation, the cells were trypsinized, suspended in fresh medium and harvested by centrifugation (1000 rpm for 5 min in room temperature). Cell pellets were then washed with cold PBS (2 × 3 mL), centrifuged (1000 rpm for 5 min) and resuspended in 100 µL of cold PBS. Subsequently, ice cold 70% ethanol was added. Tubes were left overnight at 4 °C. The fixed cells were then centrifuged (1000 rpm for 5 min). Cell pellets were rinsed with cold PBS (2 × 3 mL), centrifuged (1000 rpm for 5 min) and resuspended in the 38 mM sodium citrate with 10 µg/mL propidium iodide and 120 µg/mL DNAse-free RNAse A (AppliChem, Darmstadt, Germany). Cells were incubated in darkness for 30 min. Phases of the cell cycle were analyzed using FACSCanto II flow cytometer (Becton-Dickinson, Franklin Lakes, NJ, USA) and calculated with a use of the FACSDiva software (ver. 6.1.3, BD Biosciences Systems, San Diego, CA, USA). All experiments were conducted in duplicate in three different cultures.

### 4.5. Total Protein Content in Cells

In order to measure protein concentration in cell lysates, BCA Protein Assay Kit (Rockford, IL, USA) was used [63]. Bovine serum albumin was used as a standard. Concentrations of albumin standard: 25; 50; 100; 150; 200; 300; 400 μg/mL.

### 4.6. Western Blot Analysis

The malignant melanoma cells were rinsed with cold PBS (pH 7.4) and then solubilized in 200 μL of RIPA buffer with protease-/phosphatase-inhibitor cocktail. Obtained lysates were centrifuged (10,000× *g*, 4 °C, for 15 min), and the samples containing 10 μg of protein were subjected to SDS-PAGE, according to Laemmli [64]. The 10% polyacrylamide gel and constant current (25 mA) were used.

The proteins had been transferred to PVDF membranes and treated with blocking buffer (5% non-fat dry milk in Tris-buffered saline (TBS) containing 0.05% Tween 20 (TBS-T)) for 1 h. Subsequently, the membranes were rinsed with TBS-T and probed with appropriate antibody in 5% non-fat dry milk in TBS (concentration 1:1000). Then, membranes were left overnight at 4 °C. After incubation, membranes were washed with TBS-T. The HRP conjugated with anti-human secondary antibody against rabbit IgG at 1:1000 dilution in TBS was added. After 2 h, the membranes were rinsed with TBS-T and exposed to electrochemiluminescence (ECL) reagent. All experiments were conducted in three different cultures.

### 4.7. Chemiluminescence Detection

In order to conduct chemiluminescence detection, membranes were incubated with a luminol-based enhanced chemiluminescence substrate for peroxidase. The luminescent signal was recorded and quantified with the GeneGnome (Syngene, Frederick, MD, USA) and then detected and transmitted to the GeneTools software version 4.3.8.0 (Syngene, Frederick, MD, USA) for analysis and documentation.

### 4.8. Statistical Analysis

For statistical analysis, GraphPad Prism software version 9 (GraphPad Software, San Diego, CA, USA) was used. Data were presented as means ± standard deviations (SD). Data were statistically analyzed by ANOVA followed by Tukey’s/Dunnett’s post hoc *t*-test analysis. The significant differences in means were determined at the level of * *p* < 0.05 or ** *p* < 0.001.

## 5. Conclusions

The gathered findings suggest that MZB acts on melanoma cells in two ways: on the one hand, inducing ER stress, and on the other hand, activating the P53/P21 pathway. These actions ultimately lead to apoptotic cell death or to the induction of cellular senescence in melanoma cells. It should be taken into account that, according to the literature, maintaining the senescence state requires a high level of P16 protein, which is absent in researched melanoma cell lines. Despite the features of late senescence, at the moment, we do not know whether the senescence state induced by MZB in the G361 line is irreversible. For this reason, we consider it important to add senolytic therapy. Marizomib is better tolerated by normal cells than by melanoma cells, as we presented in our previous work [47]. We have also demonstrated its strong proapoptotic effect on malignant melanoma cells. In the current research, we presented that the effects of its activity are also visible after MZB withdrawal and are manifested by cell death or cellular senescence. Due to the lack of P16 protein and possible adverse effects of SASP, it would be reasonable to remove senescent cells with senolytics. We suggest that the combination of marizomib treatment with senolytic therapy may provide promising results in the therapy of malignant melanoma.

## Data Availability

The original contributions presented in this study are included in the article. Further inquiries can be directed to the corresponding author.
